# Persistence with Early-Line Abatacept versus Tumor Necrosis Factor-Inhibitors for Rheumatoid Arthritis Complicated by Poor Prognostic Factors

**DOI:** 10.36469/jheor.2021.23684

**Published:** 2021-05-19

**Authors:** Xue Han, Francis Lobo, Michael S. Broder, Eunice Chang, Sarah N. Gibbs, David J. Ridley, Irina Yermilov

**Affiliations:** 1 Bristol-Myers Squibb Company, Health Economics and Outcomes Research, Princeton, NJ; 2 Partnership for Health Analytic Research (PHAR), LLC, Beverly Hills, CA; 3 Saint Paul Rheumatology, Eagan, MN

**Keywords:** tumor necrosis factor inhibitors, therapy persistence, abatacept, rheumatoid arthritis, disease modifying antirheumatic drugs

## Abstract

**Background:** Rheumatoid arthritis (RA) is a chronic inflammatory disease characterized by joint swelling and destruction that leads to severe disability. There are no clear guidelines regarding the order of therapies. Gathering data on treatment patterns outside of a clinical trial setting can provide useful context for clinicians.

**Objectives:** To assess real-world treatment persistence in early-line abatacept versus tumor necrosis factor-inhibitors (TNFi) treated patients with RA complicated by poor prognostic factors (including anti-cyclic citrullinated peptide antibodies [ACPA] and rheumatoid factor [RF] seropositivity).

**Methods:** We performed a multi-center retrospective medical record review. Adult patients with RA complicated by poor prognostic factors were treated with either abatacept or TNFis as the first biologic treatment at the clinic. Poor prognostic factors included ACPA+, RF+, increased C-reactive protein levels, elevated erythrocyte sedimentation rate levels, or presence of joint erosions. We report 12-month treatment persistence, time to discontinuation, reasons for discontinuation, and risk of discontinuation between patients on abatacept versus TNFi. Select results among the subgroup of ACPA+ and/or RF+ patients are presented.

**Results:** Data on 265 patients (100 abatacept, 165 TNFis) were collected. At 12 months, 83% of abatacept patients were persistent versus 66.1% of TNFi patients (*P*=0.003). Median time to discontinuation was 1423 days for abatacept versus 690 days for TNFis (*P*=0.014). In adjusted analyses, abatacept patients had a lower risk of discontinuing index treatment due to disease progression (0.3 [95% confidence interval (CI): 0.1-0.6], *P*=0.001). Among the subgroup of ACPA+ and/or RF+ patients (55 abatacept, 108 TNFis), unadjusted 12-month treatment persistence was greater (83.6% versus 64.8%, *P*=0.012) and median time to discontinuation was longer (961 days versus 581 days, *P*=0.048) in abatacept versus TNFi patients.

**Discussion:** Patients with RA complicated by poor prognostic factors taking abatacept, including the subgroup of patients with ACPA and RF seropositivity, had statistically significantly higher 12-month treatment persistence and a longer time to discontinuation than patients on TNFis.

**Conclusions:** In a real-world setting, RA patients treated with abatacept were more likely to stay on treatment longer and had a lower risk of discontinuation than patients treated with TNFis.

## BACKGROUND

Rheumatoid arthritis (RA) is a chronic inflammatory disease characterized by joint swelling and destruction that leads to severe disability; it affects approximately 0.5-1% of the population in Europe and North America.^1–3^ Disease progression can be more rapid in patients with poor prognostic factors, which include high disease activity (increased C-reactive protein levels and elevated erythrocyte sedimentation rate levels), the presence of joint erosions, and autoantibody positivity (positive anti-cyclic citrullinated peptide antibodies [ACPA+] and positive rheumatoid factor antibodies [RF+]).^1,4–7^ ACPA and RF seropositivity precede clinical manifestations and may have an amplifying effect on inflammation and autoimmunity.^6^ ACPA seropositivity has been found to predict development of aggressive RA, resulting in higher economic burden, health-care resource utilization, and prescription costs.^8–10^

Treatment of RA usually begins with conventional, or “traditional,” disease-modifying antirheumatic drugs (DMARDs), including methotrexate, sulfasalazine, and others. Patients with an inadequate response to nonbiologic DMARDs often progress to biologic DMARDs, including tumor necrosis factor-inhibitors (TNFis), IL-6 receptor antagonists (i.e., tocilizumab and sarilumab), the anti-CD20 monoclonal antibody rituximab, and T-cell co-stimulators such as abatacept.^11–13^ TNFis (adalimumab, etanercept, infliximab, golimumab, or certolizumab pegol and their biosimilars) bind to cytokine TNF and inhibit its interaction with TNF receptors.^14^ Abatacept is a fusion protein that inhibits T-cell activation and proliferation as well as B-cell immunological response, resulting in normalization of inflammatory mediators.^15^

There are no clear guidelines regarding the order of therapies in patients failing traditional DMARDs. Instead, treatment recommendations are based on individual disease activity, and regular monitoring of patients is encouraged so treatments can be changed if disease activity does not improve or increases.^13,16,17^ Two systematic reviews compared biologic DMARDs and found similar efficacy;^18,19^ however, limited direct comparisons among the therapies were made, and populations were heterogeneous. In randomized controlled trials, abatacept has been shown to reduce disease activity and have a more acceptable safety and tolerability profile than adalimumab and infliximab.^20,21^ Additionally, there is some evidence that abatacept is associated with improved persistence and efficacy in patients with poor prognostic factors, including ACPA and RF positivity.^22–24^ Gathering data on treatment patterns outside of a clinical trial setting, including how patients switch between medications and whether patients with poor prognostic factors on abatacept are more persistent, can provide useful context for clinicians.

## OBJECTIVE

In this study, we conducted an observational, retrospective medical chart review to assess real-world treatment persistence and reasons for medication discontinuation in early-line abatacept versus TNFi treated patients with rapidly progressing RA complicated by poor prognostic factors.

## METHODS

### Study Design and Setting

We performed a multicenter retrospective medical record review of adult patients with RA at six US clinics located in Georgia, Idaho, Minnesota, North Carolina, South Carolina, and Washington. Three clinics were specialized rheumatology practices with one to four rheumatologists, and three were large, multi-specialty clinics; all treated a large volume of patients with RA and had previously participated in clinical research.

Eligible patients included adults with RA and at least one of the following poor prognostic factors: increased C-reactive protein levels, elevated erythrocyte sedimentation rate levels, the presence of joint erosions, ACPA+, or RF+. Our goal was to include patients who had received abatacept or a TNFi as their first biologic treatment. To meet this goal in a real-world population, we included patients whose first drug at the study site was either abatacept or a TNFi, even if they may have been treated with other RA drugs at other clinics, resulting in a significant limitation of the study. We defined this study population as having evidence of early-line abatacept or TNFi use. To be eligible for inclusion, patients had to have been treated with either abatacept or a TNFi as their first biologic treatment at the clinic, and this treatment had to be initiated on or after July 31, 2011. This date was chosen because it was the FDA approval date for abatacept (e.g., all included patients received their first biologic at the clinic—abatacept or TNFis—after abatacept approval). TNFis included adalimumab, etanercept, infliximab, golimumab, or certolizumab pegol. Patients were excluded if they had Crohn’s disease, ankylosing spondylitis, ulcerative colitis, psoriatic arthritis, or anal fistula. Data abstractors found eligible patients by first identifying the date and type of first line biologic treatment then applying the inclusion and exclusion criteria.

In an initial attempt to collect data on 400 patients across six sites with balanced study arms, the sample goal of enrolled patients was 33 TNFi and 34 abatacept patients per site. A random number generator was used to identify a subset if the abstractor found more patients than could be enrolled. However, as recruitment progressed, fewer patients were identified than needed, so all eligible patients were enrolled at some sites and a few sites enrolled either more abatacept or TNFi patients. The data were collected between March 2018 through October 2019. Attrition (including number of patients excluded based on study criteria) was not documented.

The study was approved by a central Institutional Review Board (Western IRB, Tracking number 20172723). The Board found that this research meets the requirements for a waiver of consent under 45 CFR 46.116(d).

### Data Collection/Variables

A secure web-based electronic case report form (eCRF) was designed in collaboration with physician investigators from participating clinics. Each clinic identified one to two abstractors to review patient medical records and enter data into the eCRF. The abstractors included research coordinators and nurses. Research staff trained abstractors to apply inclusion/exclusion criteria and accurately enter data using a de-identified medical record from one of the participating clinics. Abstractors were only able to begin data collection using the eCRF after they satisfactorily completed the training.

**Figure 1.**
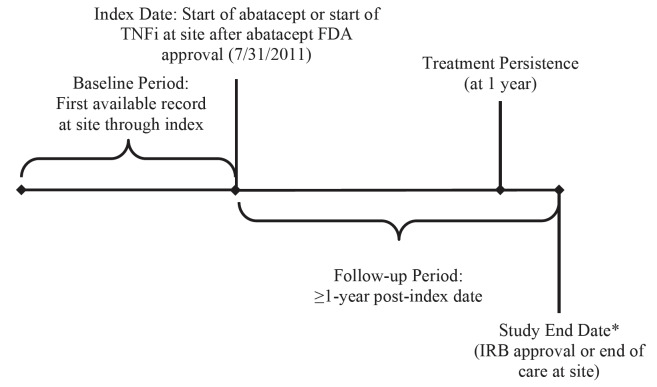
Study Design Abbreviations: FDA, Food and Drug Administration; IRB, Institutional Review Board; TNFi, tumor necrosis factor-inhibitors. *In a retrospective study, data usually cannot be collected after IRB approval.

Data were collected from the index date (the start of abatacept or TNFis at the site) for at least one year through the IRB approval date or the end of care at the site, whichever came first (Figure 1). Baseline data were collected from the first record of care at the clinic through the index date. Data collected included demographics (sex, birth year), presence of baseline comorbidities, and baseline disease history (duration of RA, treatment at the clinic). The primary outcome measure was persistence with treatment, defined as the duration of time from initiation to discontinuation of therapy, consistent with the International Society for Pharmacoeconomics and Outcomes Research Medication Adherence and Persistence Special Interest Group definition.^25^ Duration of index treatment was calculated as the time between when the index treatment started and when the index treatment stopped. Treatment gaps of ≤ 60 days were ignored. Other outcomes included health-care utilization (office visits and hospitalizations one year prior to the index date) and reason for treatment discontinuation (including as a result of disease progression) as recorded in the medical record.

Each record was reviewed for errors and logical consistency. The eCRF included automatic validity checks (e.g., invalid or illogical dates). Research staff also conducted regular data quality checks for content, inconsistencies, and missing fields. Inconsistent data and potential errors were flagged by research staff and verified with the site abstractors. Missing data were confirmed to be missing (rather than omitted in error) by the site abstractors. The only variable with missing data was the reason for discontinuation of the index medication. Less than 2% of the sample were missing this variable; these patients were included in the adjusted analysis as part of the cohort that did not discontinue the index medication for disease progression.

### Data Analysis

Unadjusted analyses of the patient demographics, comorbidities, disease history, health-care utilization, and reasons for treatment discontinuation were conducted. Chi-square tests (or exact chi-square tests for a cell count <5) and t-tests were performed for categorical variables and continuous variables, respectively. Treatment persistence (continuation of index treatment with gap ≤60 days) at 12 months and time to discontinuation were calculated. Select unadjusted analyses were performed post-hoc among the subgroup of ACPA+ and/or RF+ patients.

Multivariate logistic and Cox proportional hazards regression models were used to compare 12-month persistence and risk of discontinuation between abatacept and TNFi patients, controlling for demographic and clinical characteristics (sex, age, Charlson comorbidity index [CCI], duration of RA), health-care utilization, and clinic. All analyses were conducted in SAS version 9.4 (Cary, NC).

## RESULTS

Data on 265 patients (100 abatacept, 165 TNFi) were collected (Table 1). Most TNFi patients were either taking adalimumab (40.6%) or etanercept (33.3%). The percentage of abatacept and TNFi patients differed by clinic site. Patients on abatacept were older than those taking TNFis (67.0 vs 60.3 years, *P*<0.001). Abatacept patients had more pre-index hospitalizations. There were no other significant differences in sex, CCI, duration of RA, health-care utilization, or duration of treatment at the clinic. Among the subgroup of ACPA+ and/or RF+ patients, 55 were on abatacept and 108 were on TNFis (Table 1). The subgroup had similar demographic and clinical characteristics to the full cohort.

**Table 1.** Baseline Characteristics

	**All Patients**			**ACPA+ and/or RF+ Patients**		
	**Abatacept n=100**	**TNFi n=165**	***P*﻿-﻿Value**	**Abatacept n=55**	**TNFi n=108**	***P*﻿-﻿Value**
**TNFi, n (%)**			n/a			n/a
Adalimumab	-	67 (40.6)		-	44 (40.7)	
Etanercept	-	55 (33.3)		-	36 (33.3)	
Infliximab	-	28 (17.0)		-	20 (18.5)	
Golimumab	-	11 (6.7)		-	6 (5.6)	
Certolizumab Pegol	-	4 (2.4)		-	2 (1.9)	
**Clinic, n (%)**			<0.001			0.002
1	5 (5.0)	33 (20.0)		5 (9.1)	27 (25.0)	
2	24 (24.0)	42 (25.5)		16 (29.1)	30 (27.8)	
3	45 (45.0)	22 (13.3)		16 (29.1)	12 (11.1)	
4	10 (10.0)	51 (30.9)		6 (10.9)	27 (25.0)	
5	5 (5.0)	11 (6.7)		4 (7.3)	6 (5.6)	
6	11 (11.0)	6 (3.6)		8 (14.6)	6 (5.6)	
**Female, n (%)**	82 (82.0)	121 (73.3)	0.106	46 (83.6)	78 (72.2)	0.106
**Age in Years (in 2017), Mean (SD)**	67.0 (13.6)	60.3 (12.2)	<0.001	64.5 (12.7)	59.4 (12.5)	0.014
**CCI, Mean (SD)**	0.7 (1.0)	0.5 (0.9)	0.080	0.7 (1.1)	0.5 (0.9)	0.170
**Total Duration of Treatment at Clinic (years), Mean (SD)**	5.7 (4.2)	5.2 (3.4)	0.288	5.1 (3.7)	4.8 (3.0)	0.575
**No. Physician Office Visits (1-year pre-index), Mean (SD)**	3.8 (2.4)	4.0 (3.4)	0.588	3.7 (2.2)	3.6 (2.3)	0.760
**No. Hospitalizations (1-year pre-index), Mean (SD)**	0.1 (0.4)^a^	0.1 (0.3)^a^	0.037	0.2 (0.4)^b^	0.1 (0.4)^b^	0.084

Abbreviations: ACPA+, positive anti-cyclic citrullinated peptide antibodies; CCI, Charlson comorbidity index; RF+, positive rheumatoid factor antibodies; SD, standard deviation; TNFi, tumor necrosis factor inhibitor.

^a^ Additional detail provided to describe significant differences: abatacept=0.12 (0.35); TNFi=0.06 (0.29).

^b^ Additional detail provided to describe significant differences: abatacept=0.18 (0.43); TNFi=0.10 (0.36).

**Figure 2.**
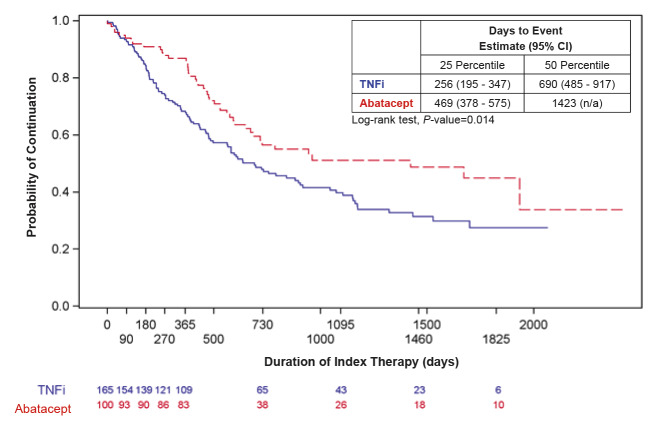
Time to Discontinuation of Index Treatment Among All Patients (N=265) Abbreviations: CI, confidence interval; N/A, not applicable; TNFi, tumor necrosis factor inhibitor. Blue solid line=TNFi; Red dashed line=abatacept

**Figure 3.**
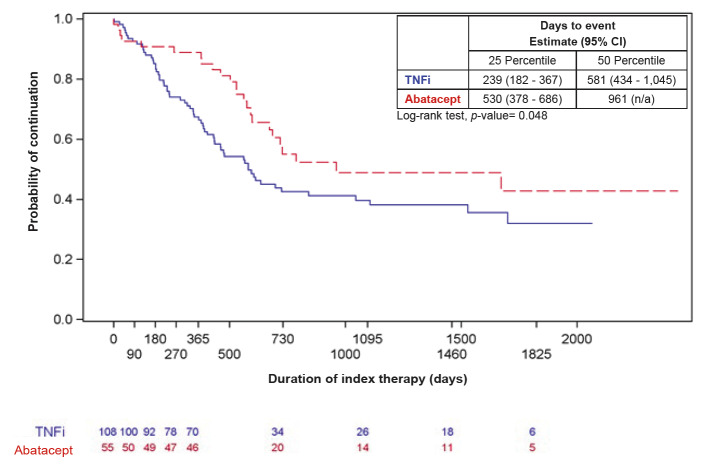
Time to Discontinuation of Index Treatment Among ACPA+ and/or RF+ Patients (n=163) Abbreviations: ACPA+, positive anti-cyclic citrullinated peptide antibodies; CI, confidence interval; N/A, not applicable; RF+, positive rheumatoid factor antibodies; TNFi, tumor necrosis factor inhibitor. Blue solid line=TNFi; Red dashed line=abatacept

In unadjusted analyses, patients on abatacept had statistically significantly higher treatment persistence at 12 months than patients on TNFis (83% vs 66.1%, *P*=0.003) (Table 2). Median time to discontinuation of index treatment was 1423 days for abatacept versus 690 days for TNFis (*P*=0.014) (Figure 2). Findings were similar among the subgroup of ACPA+ and/or RF+ patients: Treatment persistence at 12 months was greater in patients on abatacept versus TNFis (83.6% vs 64.8%, *P*=0.012) and median time to discontinuation was longer in patients on abatacept versus TNFis (961 days vs 581 days, *P*=0.048) (Figure 3).

**Table 2.** Treatment Persistence and Reason for Discontinuation

	**All Patients**			**ACPA+ and/or RF+ Patients**		
	**Abatacept n=100**	**TNFi n=165**	***P*﻿-﻿Value**	**Abatacept n=55**	**TNFi n=108**	***P*﻿-﻿Value**
**Index Drug with 12 Months of Persistence, n (%)**	83 (83.0)	109 (66.1)	0.003	46 (83.6)	70 (64.8)	0.012
**Reason for Discontinuation (among patients who discontinued index treatment), n (%)**			<0.001			0.007
Disease Progression (uncontrolled symptoms or on laboratory testing)	12 (26.7)	45 (44.1)		5 (20.8)	31 (49.2)	
Adverse Effects of Medication	1 (2.2)	13 (12.8)		0 (0)	9 (14.3)	
Insurance Coverage	8 (17.8)	15 (14.7)		6 (25.0)	7 (11.1)	
Adherence Issues	1 (2.2)	0 (0)		1 (4.2)	0 (0)	
Physician Preference	1 (2.2)	5 (4.9)		1 (4.2)	3 (4.8)	
Patient Preference	4 (8.9)	6 (5.9)		3 (12.5)	2 (3.2)	
Other Reasons	14 (31.1)	18 (17.7)		7 (29.2)	11 (17.5)	
Unknown/Not Specified	4 (8.9)	0 (0)		1 (4.2)	0 (0)	

Abbreviations: ACPA+, positive anti-cyclic citrullinated peptide antibodies; RF+, positive rheumatoid factor antibodies; TNFi, tumor necrosis factor inhibitor.

Reasons for discontinuation of index treatment differed between the two cohorts (Table 2). More patients on abatacept discontinued treatment due to insurance coverage while more patients on TNFis discontinued treatment due to disease progression or adverse effects. Findings were similar among the subgroup of ACPA+ and/or RF+ patients.

In the logistic regression model, the odds of abatacept patients being persistent at 12 months were 1.980 compared to TNFi patients, although the difference was not statistically significant (95% CI 0.943-4.167, *P*=0.071) (Table 3). In the Cox proportional hazards model, risk of all-cause discontinuation was significantly lower among patients on abatacept than those on TNFis (Hazard Ratio [HR] 0.584, 95% CI 0.384-0.887, *P*=0.012) (Table 3). Patients on abatacept also had a statistically significantly lower risk of discontinuing index treatment due to disease progression (HR 0.293, 95% CI 0.138-0.620, *P*=0.001) (Table 3).

**Table 3.** Adjusted Treatment Persistence and Risk of Discontinuation Among All Patients (N=265)^a^

	**Persistence at 12 Months: OR (95% CI)**	***P*﻿-﻿Value**	**Risk of All-Cause Discontinuation: HR (95% CI)**	***P*﻿-﻿Value**	**Risk of Discontinuation for Disease Progression**	***P*﻿-﻿Value**
**Clinic**						
1 vs. 6	3.208 (0.839 - 12.275)	0.089	0.459 (0.214 - 0.985)	0.046	0.472 (0.144 - 1.547)	0.215
2 vs. 6	1.679 (0.522 - 5.396)	0.385	0.481 (0.242 - 0.957)	0.037	0.484 (0.163 - 1.436)	0.191
3 vs. 6	14.754 (3.440 - 63.274)	<0.001	0.286 (0.144 - 0.569)	<0.001	0.192 (0.060 - 0.615)	0.006
4 vs. 6	3.656 (1.076 - 12.425)	0.038	0.299 (0.146 - 0.614)	0.001	0.016 (0.002 - 0.150)	<0.001
5 vs. 6	1.762 (0.397 - 7.809)	0.456	0.562 (0.236 - 1.339)	0.194	0.930 (0.273 - 3.167)	0.908
**Male vs. Female**	0.508 (0.255 - 1.010)	0.053	1.216 (0.826 - 1.791)	0.321	1.280 (0.688 - 2.380)	0.435
**Age, Years**	1.013 (0.988 - 1.039)	0.319	1.002 (0.988 - 1.016)	0.803	0.996 (0.973 - 1.019)	0.711
**CCI**	1.003 (0.731 - 1.377)	0.985	1.012 (0.842 - 1.216)	0.898	0.982 (0.730 - 1.321)	0.903
**Years from RA Diagnosis**	0.988 (0.951 - 1.027)	0.540	1.010 (0.987 - 1.033)	0.408	1.018 (0.981 - 1.056)	0.341
**No. Physician Office Visits (1-year pre-index)**	Not significant		Not significant		Not significant	
**No. Hospitalizations (1-year pre-index)**	Not significant		Not significant		Not significant	
**Abatacept vs TNFi**	1.980 (0.943 - 4.167)	0.071	0.584 (0.384 - 0.887)	0.012	0.293 (0.138 - 0.620)	0.001

Abbreviations: CCI, Charlson comorbidity index; CI, confidence interval; HR, hazard ratio; OR, odds ratio; RA, rheumatoid arthritis; TNFi, tumor necrosis factor inhibitor.

^a^ The initial models included age, sex, CCI, years from RA diagnosis to index, and cohort as independent variables. We then used a forward selection method to include additional significant covariates (*P*<0.05) for the final models. The following covariates were considered: number of physician office visits (1-year pre-index), number of hospitalizations (1-year pre-index), and site.

## DISCUSSION

In this study’s real-world setting, patients with RA complicated by poor prognostic factors taking abatacept had statistically significantly higher 12-month treatment persistence and a significantly longer time to discontinuation than patients on TNFis. Among the subgroup of ACPA+ and/or RF+ patients, patients on abatacept also showed significantly higher 12-month persistence and longer time to discontinuation.

Our results are consistent with outcomes from clinical trials, although the trials made different comparisons than our study. In a post hoc analysis of AMPLE, the authors reported that in patients with early RA and poor prognostic factors, abatacept showed a trend toward greater efficacy compared with adalimumab.^26^ Earlier AMPLE analyses had demonstrated fewer abatacept patients discontinued therapy due to adverse events than adalimumab patients.^27^ In the AGREE trial, patients with rapidly-progressing RA and poor prognostic factors who received abatacept plus MTX had significantly better clinical outcomes compared with MTX alone.^28^ In these and other trials, abatacept has been shown to improve disease activity and quality of life among patients who remained on the treatment longer.^20,25,28^ Similarly, the PREMIER study found that in patients with early, aggressive RA, combination therapy with adalimumab plus MTX was significantly better than either MTX alone or adalimumab alone in improving signs and symptoms of disease.^29^

Some of our results are also similar to observational studies. A recent retrospective study in Canada compared persistence with abatacept and TNFis used as a first-line biologic and found similar persistence at nine years. As a second-line biologic agent, abatacept had better persistence rates than TNFis.^27^ Another retrospective cohort study in South Korea also found abatacept had higher persistence (60.4%) compared to adalimumab (45.7%), etanercept (58.5%), and infliximab (43.0%).^30^ A US health-care claims analysis study found patients were more likely to be persistent on abatacept, tocilizumab, or tofacitinib compared to TNFis^31^ (61.8% vs 58.2%). Our subgroup analysis of patients with ACPA and RF seropositivity were also consistent with other studies: observational studies have shown higher persistence on abatacept and lower persistence on TNFis among patients with ACPA and RF seropositivity.^32,33^ These studies conclude higher persistence could be the result of better effectiveness of abatacept, or lower effectiveness of TNFis, in this population, which would also align with our findings that more patients discontinued TNFis than abatacept due to disease progression among this subgroup.

Medication persistence is key to deriving benefit from therapy and can be measured in different ways, including the time between refills, number of refills, the proportion of patients dispensed a certain number of days’ supply of medication, or the proportion of patients continuing to refill prescriptions after a specified time interval. In this study, we used a measure of persistence consistent with the International Society for Pharmacoeconomics and Outcomes Research Medication Adherence and Persistence Special Interest Group: “the duration of time from initiation to discontinuation of therapy.”^25^ Persistence is a less ambiguous measure of medication-taking behavior than “adherence,” which typically means the extent to which a patient takes the correct dose at the correct interval. Clinically, persistence with therapy usually indicates that the disease has not progressed and that the medication is tolerable.

This study had several limitations. First, the study was not adequately powered to detect differences in disease outcomes. Second, the study was retrospective and differences in persistence between cohorts may be the result of unmeasured differences in patient characteristics, insurance coverage, or provider practices. Patients were not equally distributed across cohorts at every clinic, and each clinic did not enroll the same number of patients. Further, one clinic reported higher persistence across all patients than others and clinics varied in size (specialty rheumatology practices versus multi-specialty clinics) and geography (Georgia, Idaho, Minnesota, North Carolina, South Carolina, and Washington), which may have affected provider practice. Abatacept, adalimumab, and etanercept may all be self-administered, and medical records may overstate adherence, as they are based on patient report.^34^ Indeed, patients on abatacept in our study had higher 1-year persistence than has been reported in administrative claims studies,^35,36^ and we found higher persistence in all patients compared to other observational studies.^30,37^ This finding may be because our study required at least one year of data post index date for all patients, which may have artificially inflated the duration of follow-up and persistence and potentially lowered the external validity of the findings. We have no reason to believe that the persistence rates of the abatacept and TNFi cohorts were differentially affected by any reporting errors. We defined early-line treatment as the first treatment provided at the clinic, but patients may have been treated for RA with other drugs before starting treatment at study sites, resulting in a significant real-world limitation. However, based on prior treatments reported in the eCRF, we estimate this occurred in only a small number of patients (approximately 13%). Lastly, we did not collect data on attrition (e.g., the number of patients excluded based on study criteria).

## CONCLUSIONS

This study provides quantitative and qualitative data about how patients with RA and poor prognostic factors are treated. In a real-world setting, patients on abatacept, including the subgroup of patients with ACPA and RF seropositivity, stayed on treatment longer and had a lower risk of discontinuation due to disease progression than patients on TNFis.
